# Prevalence of osteoporosis in spinal surgery patients older than 50 years: A systematic review and meta-analysis

**DOI:** 10.1371/journal.pone.0286110

**Published:** 2023-05-25

**Authors:** Zhi-qiang Fan, Xin-an Yan, Bao-feng Li, Erdong Shen, Xin Xu, Hu Wang, Yan Zhuang

**Affiliations:** Department of Pelvic and Acetabular Surgery, HongHui Hospital, Xi’an Jiaotong University, Xi’an, China; Mie University Graduate School of Medicine, JAPAN

## Abstract

**Introduction:**

In spine surgery, poor bone condition is associated with several complications like adjacent segment fractures, proximal junctional kyphosis, and screw loosening. Our study explored the prevalence of osteoporosis in spinal surgery patients older than 50 years through a systematic review and meta-analysis.

**Methods:**

This systematic review and meta-analysis were conducted according to the PRISMA criteria. Three electronic databases, including PubMed, EMBASE, and Web of Science, were searched from inception to August 2022. We used the random-effects model to calculate the overall estimates, and the heterogeneity was measured using Cochran’s Q and *I*^2^ tests. Meta-regression and subgroup analyses were used to determine the source of the heterogeneity.

**Results:**

Based on the inclusion and criteria, we chose ten studies with 2958 individuals for our analysis. The prevalence of osteoporosis, osteopenia, and osteoporosis/osteopenia in the spinal surgery patients was 34.2% (95%CI: 24.5%–44.6%), 43.5% (95%CI: 39.8%–47.2%), and 78.7% (95%CI: 69.0%–87.0%), respectively. Regarding different diagnoses, the prevalence was highest in patients with lumbar scoliosis (55.8%; 95%CI: 46.8%-64.7%) and the lowest in patients with cervical disc herniation (12.9%; 95%CI: 8.1%-18.7%). In age groups 50–59, 50–69,70–79, the prevalence was 27.8%, 60.4%, 75.4% in females, and 18.9%, 17.4%, 26.1% in males.

**Conclusions:**

This study showed a high prevalence of osteoporosis in patients undergoing spine surgery, especially in females, people of older age, and patients who received degenerative scoliosis and compression fractures. Current osteoporosis screening standards for patients undergoing spine surgery may not be adequate. Orthopedic specialists should make more efforts regarding preoperative osteoporosis screening and treatment.

## Introduction

Osteoporosis is a common disease in the elderly, characterized by a decrease in bone mass and an increased risk of fragility fractures [1]. In adults aged> 50 years, the global prevalence of osteoporosis was reported as 20.5% [2]. Twenty-two million women and 5.5 million men were estimated to experience osteoporosis in Europe in 2010, causing 3.5 million fragility fractures and a €37 billion economic burden annually [3].

As the global population ages, more people are required to receive spine surgery, which burdens society heavily [4]. In spine surgery, osteoporosis has been considered to be linked with several complications, including adjacent segment fractures, screw loosening, and proximal junctional kyphosis [5, 6]. For instance, Bone mineral density (BMD) is a key determinant of screw fusion rates in spine surgery. A lower spine BMD could result in a long recovery time for patients who experienced fusion procedures with instrumentation [7]. Previous studies showed that osteoporosis was highly prevalent in spinal surgery patients [8, 9]. In a cohort of 104 spine surgery candidates, Anderson PA et al. [8] reported 48 patients (46%) with osteoporosis and 50 patients (48%) with osteopenia when using WHO (World health organization) diagnosis criteria (osteopenia as -2.5 < T-score < -1.0, osteoporosis as T-scores< -2.5). In a retrospective study conducted by Chin DK et al. [9], osteoporosis was found in 51.4% of females and 14.5% of males undergoing spine surgery. Strategies like drug treatment, the use of cement augmentation of pedicle screws, multiple points fixations, and the newly designed pedicle screw have been carried out by surgeons to address the issues [10, 11]. In a prospective study in Japan, S. Ohtori et al. [12] found that teriparatide could improve the quality of the pedicle cortex and bone marrow. Nevertheless, surgeons did not pay enough attention to the bone quality of spinal surgery patients [13, 14]. A survey of surgeons showed that only 44% of instrumented fusion patients received preoperative Dual-energy Xray absorptiometry (DXA). A recommendation by the International Society for Clinical Densitometry (ISCD) suggested that surgeons should evaluate bone health in males aged ≥70 years and females aged ≥65 years who undergo spine surgery [15]. In a study of preoperative BMD assessment for spinal deformity surgery, T. K. Kuprys et al. [16] reported that the rate of DXA screening was less than the recommended guidelines. In addition, the high prevalence of osteoporosis in female patients older than 50 indicated that the ISCD recommendation might not be adequate [17, 18]. Although some studies have investigated the prevalence of osteoporosis in spinal surgery patients, there has not been a study analyzing these data. Therefore, this study aims to estimate the osteoporosis prevalence in patients undergoing spine surgery through a systematic review and meta-analysis.

## Methods

This study was conducted based on the Preferred Reporting Items for Systematic and Meta-analyses guidelines (PRISMA) [19] (PRISMA checklist; S1 Appendix).

### Search strategy

Three electronic databases (PubMed, EMBASE, and Web of Science) were chosen for searching the article that reported the osteoporosis prevalence in patients undergoing spine surgery from inception to August 2022. The search language was limited to only English. The following terms and keywords were combined for searching: “spine surgery”, “lumbar surgery”, “osteoporosis”, “osteopenia”, “bone mineral density”, “prevalence”, “incidence”, and “epidemiology.” We also conducted a hand search of references in relevant articles. The detailed search strategy is listed in S2 Appendix.

### Study eligibility

Two reviewers (FZQ, YXA) independently reviewed titles and abstracts on eligibility criteria for inclusion and then read the full article. Any discrepancies will be resolved by the discussion between two authors (FZQ, YXA) and a third reviewer (ZY). Inclusion criteria are as following: (1) Longitudinal observational studies; Cross-sectional studies (2) Studies reporting the osteoporosis prevalence in patients undergoing spine surgery (3) BMD was measured by DXA examination at femur or lumbar spine. (4) Osteoporosis and osteopenia were defined by WHO diagnosis criteria (osteopenia as -2.5 < T-score < -1.0, osteoporosis as T-scores< -2.5).

The exclusion criteria are as following: (1) Conference abstracts, reviews, letters, or comments. (2) BMD is not measured by DXA; (3) Studies with no full text or sufficient data.

### Data extraction

Two reviewers independently collected the following data: Publication year, author’s name, study period, number of females, sample size, study design, number of patients with osteoporosis and osteopenia, diagnosis method, body mass index (BMI), mean age, DXA examination sites, procedure indications, and study quality. Any disagreements between two reviewers are resolved by discussing with a third reviewer.

### Quality assessment

Each study was assessed using a quality assessment checklist developed from the ‘Risk of bias tool’ from Hoy et al. [20], which contains 10 criteria (S3 Appendix). Each criterion provides a "yes," "no," or "don’t know" response option. If the answer to a criterion is "yes," the score is "1." A "No" or "Don’t know" answer is scored as "0." Accordingly, the aggregate scores for the chart span from 0 to 10. Studies with scores between 8 to 10 are considered "low-risk," 5 to 7 are considered "moderate risk," and 0 and 4 are considered "high-risk." High-risk studies will be excluded after quality assessment.

### Statistical analysis

All analyses were performed using R software (version 4.1). A random effects model was used to calculate the integrated estimates. To stabilize the variance, we transformed the data using the Freeman-Tukey double arcsine transform. The primary outcome was the pooled prevalence of osteoporosis and osteopenia in spinal surgery patients. We used Cochran’s Q test and the *I*^*2*^ statistic to analyze heterogeneity, and *I*^*2*^≥50% was considered high heterogeneity. Subgroup analysis was also conducted as follows: Sex (female, male); Procedure indications; Age (>50, 50–59, 60–69, 70–79); Continent (Europe, Asia, and North America). Funnel plot and Egger’s test were used to measure publication bias.

## Results

### Literature search and characteristic

**Fig 1:**
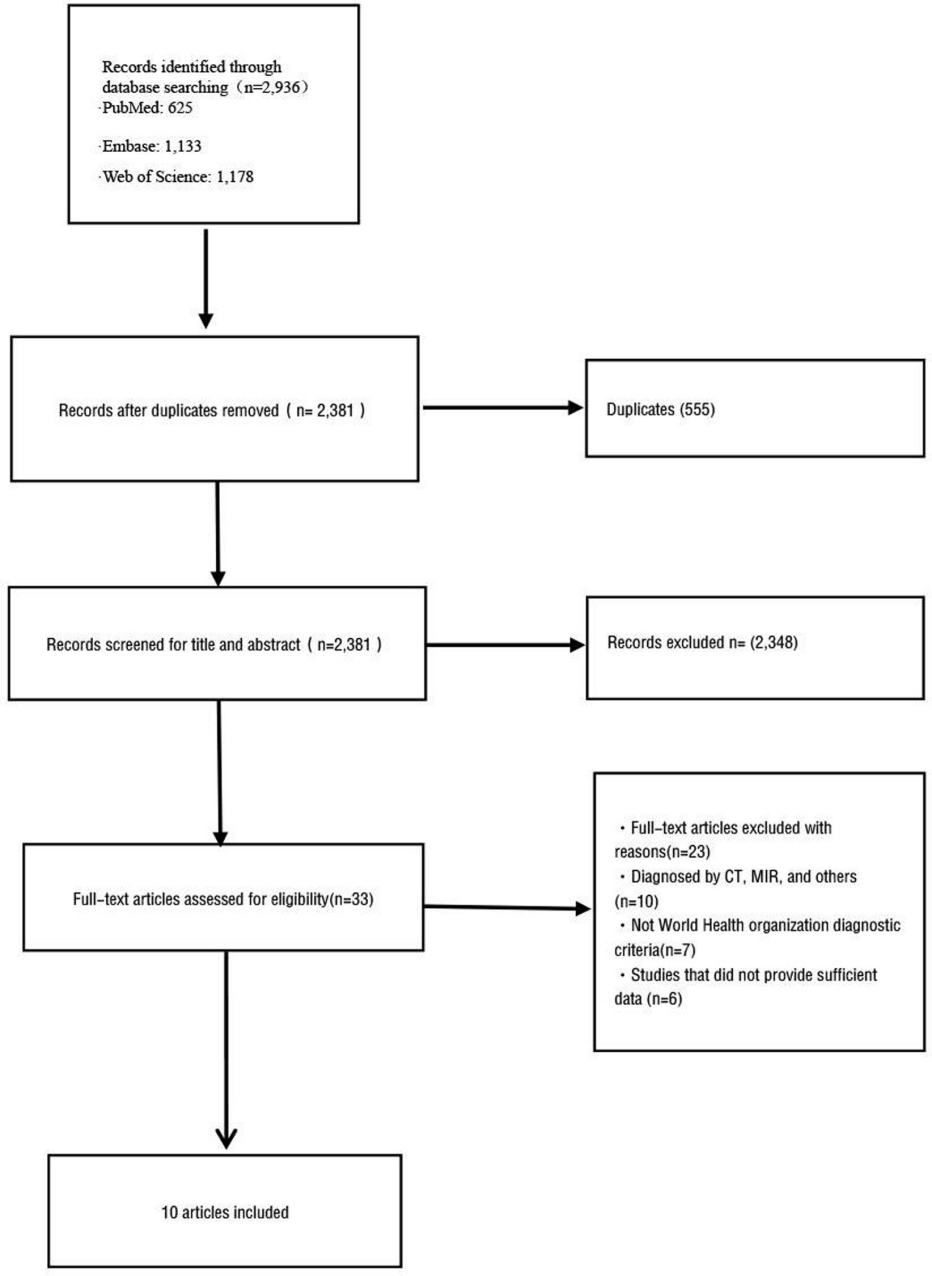
Flow diagram of identification and selection of studies for inclusion in the meta-analysis.

Fig 1 provides the flow chart of study selection. We initially searched 2,936 citations from three databases (PubMed: 625, Embase: 1,133, Web of Science: 1,178). Then, 555 citations were excluded since duplication, 2,381 citations were excluded after the title and abstract screening, and 23 were excluded after full-text reading. Finally, 10 studies met the inclusion criteria for analysis. A total of 2,958 individuals (1,764 females and 1,194 males) undergoing spine surgery were included in our study. The osteoporosis prevalence varied from 9.6% to 50.8% in included studies. Studies were carried out in eight countries including Spin [21], Germany [22], Sweden [17], France [23], China [18, 24], Korea [9], India [25], America [8, 26]. The mean age of participants in individual studies varied from 60.9 to 71.2. The detailed characteristics of included studies were shown in Table 1. After quality assessment, there were 8 low-risk studies, 2 moderate-risk studies, and no high-risk studies. The quality assessment form was presented in S4 Appendix.

**Table 1:** Characteristics of the eligible studies for this meta-analysis.

Author(et.al) Year	Study Period	Countries	Study Design	Sample Size	NO. of Female	Age (Mean (SD); Rang (years))	BMI (Mean (SD)(kg/m2))	NO. of Osteoporosis	NO. of Osteopenia	Diagnosis method	DXA examination sites	Procedure indications	Quality Score
Paz RD et al. 2022	2019	Spain	cross-sectional	104	57	60.9 (7.6), >50	31.0 (NA)	10	36	BMD measured by DXA WHO criterion	Lumbar spine (L1-4) and hips	Spondylotic lumbar stenosis, Degenerative spondylolisthesis, Herniation of cervical disc, Cervical spondylotic stenosis with myelopathy	8
Schmidt T et al. 2018	2015–2016	Germany	retrospective	144	96	70.8 (8.1), >50	26.2 (4.7)	39	63	BMD measured by DXA WHO criterion	Lumbar spine (L1-4) and hips	Lumbar spinal stenosis, Degenerative spondylolisthesis, Herniation of lumbar disc, Compression fracture	9
Bergh C et al. 2018	2013–2014, and 2016	Sweden	prospectively	65	37	67.0 (8.5), >50	28.0 (4.0)	33	23	BMD measured by DXA WHO criterion	Lumbar spine	Lumbar spinal stenosis	8
Banse C et al. 2019	2015–2017	France	retrospective	28	25	71.2 (NA), >50	30.7 (NA)	4	12	BMD measured by DXA	Lumbar spine, femoral neck and/ or ultra-distal radius	Scoliosis and spondylolisthesis	6
Zou D et al. 2020	2015–2016	China	retrospective	479	276	61.8 (6.8), >50	26.0 (3.4)	190	217	BMD measured by DXA WHO criterion	Lumbar spine (L1-4) and hips	Degenerative lumbar spinal stenosis, Lumbar disc herniation, Degenerative lumbar spondylolisthesis, Degenerative lumbar scoliosis	8
Chin DK et al. 2007	2005	Korea	retrospective	516	323	62.6 (8.0), >50	NA	194	223	BMD measured by DXA WHO criterion	Femur head and lumbar spine	Tumor, Compression fracture, Degenerative spondylolisthesis, Herniation of cervical disc, Herniation of lumbar disc, Spondylolytic spondylolisthesis, Spondylotic stenosis, Miscellaneous	9
Dave D et al. 2022	NA	India	cross-sectional	29	16	66.8 (7.9), Males ≥60 females ≥55 years	28.1 (5.2)	19	8	BMD measured by DXA WHO criterion	Femoral neck, lumbar spine, and radius	Spinal procedure	7
Mo X et al. 2021	2018–2019	China	cross-sectional	1245	678	62.2 (8.0), >50	NA	464	534	BMD measured by DXA WHO criterion	Lumbar spine (L1-L4) and hips (femoral neck and total hip).	Vertebral fracture, Degenerative stenosis, Degenerative scoliosis, Degenerative spondylolisthesis, Cervical disc herniation, Lumbar disc herniation	9
Anderson PA et al. 2020	2017–2019	America	retrospective	104	84	69.0 (8.1), >50	27.6 (5.8)	48	50	BMD measured by DXA WHO criterion	Femoral neck, lumbar spine, and radius	Thoracolumbar surgery	8
St Jeor JD et al. 2020	2007–2018	America	retrospective	244	172	68.3 (9.2), >50	28.8 (5.9)	62	132	BMD measured by DXA WHO criterion	Hips and/or spine	Lumbar degenerative pathology	9

NO = number. BMD = bone mineral density. SD = standard deviation. BMI = body mass index. NA = not applicate. DXA = Dual-energy X-ray absorptiometry. WHO = World Health Organization.

### Overall

**Fig 2:**
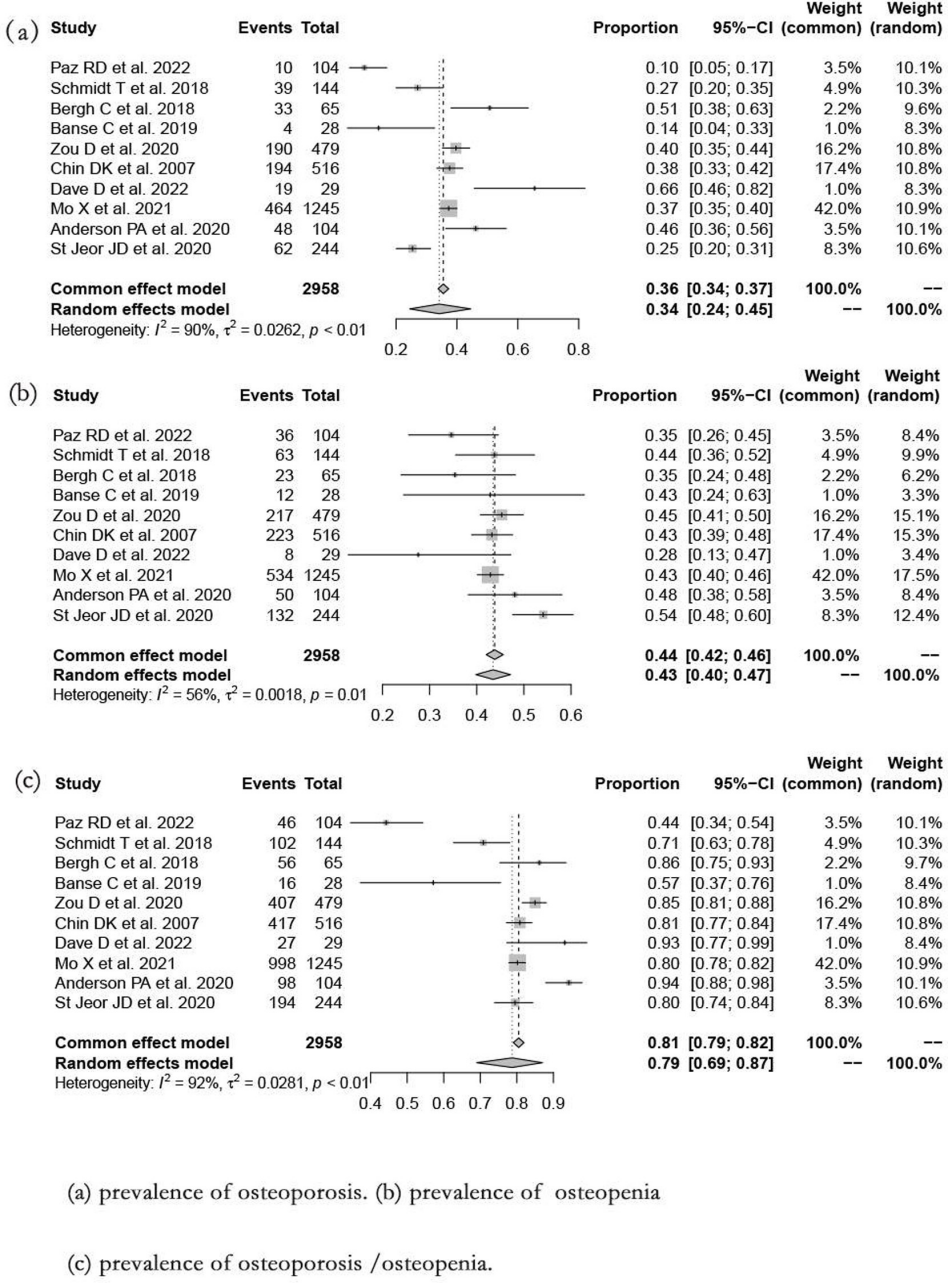
Forest plot of prevalence in patients older than 50 years undergoing spine surgery. (a) prevalence of osteoporosis. (b) osteopenia. (c) osteoporosis /osteopenia.

The overall osteoporosis prevalence in patients > 50 years undergoing spine surgery was 34.2% (95%CI: 24.5%–44.6%; *I*^*2*^ = 90.3%; *P*<0.01) (Fig 2A). The osteopenia prevalence was 43.5% (95%CI: 39.8%–47.2%; *I*^*2*^ = 56.3%; *P* = 0.01) (Fig 2B). The osteoporosis/osteopenia prevalence was 78.7% (95%CI: 69.0%–87.0%; *I*^*2*^ = 91.5%; *P*<0.01) (Fig 2C).

### Sex- and age-specific groups

**Fig 3:**
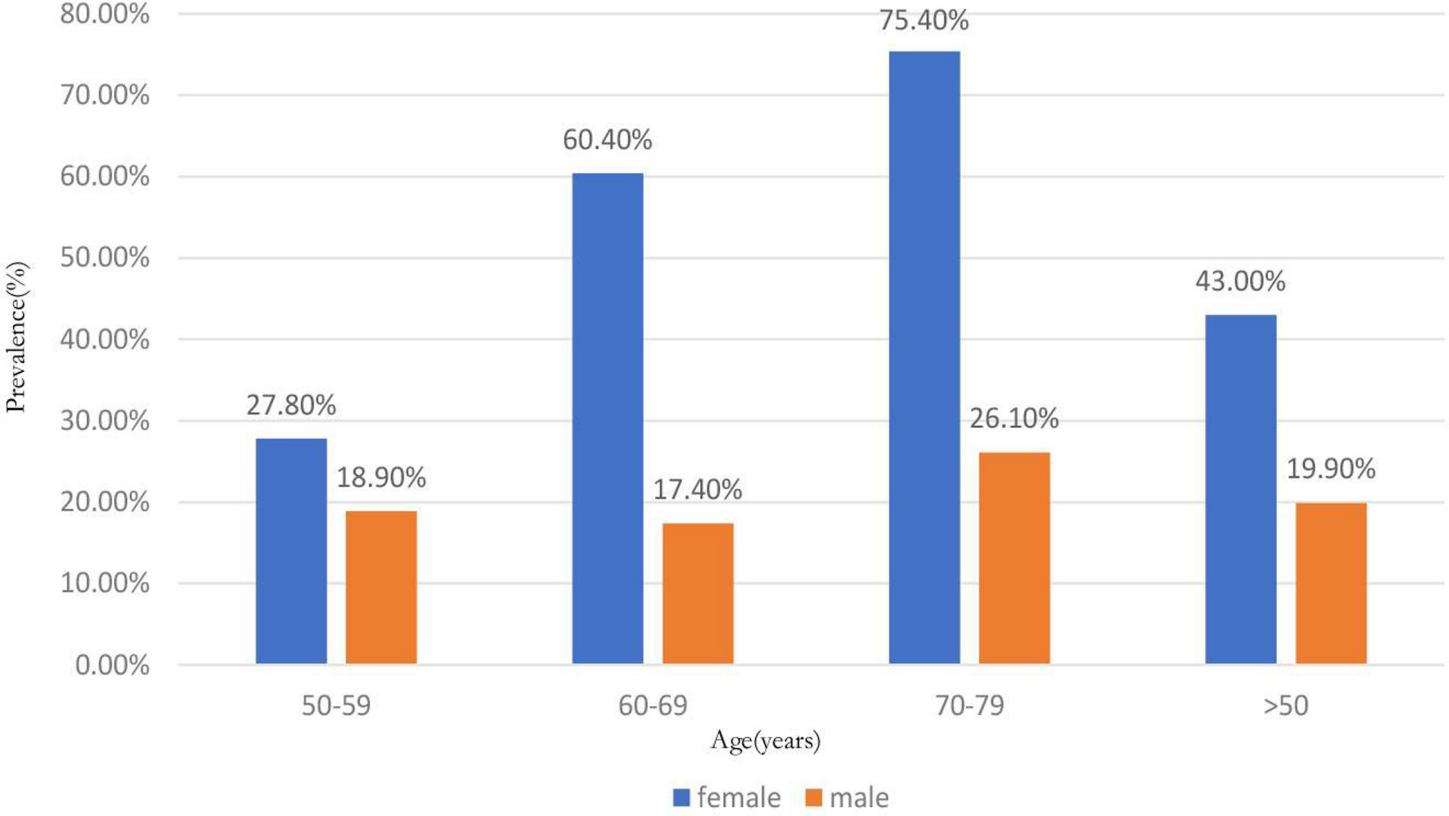
Age- and sex-specific prevalence of osteoporosis in patients undergoing spine surgery.

The osteoporosis prevalence in male and female were 19.9% (95%CI: 9.1%–33.6%; *I*^*2*^ = 86.6%; *P*<0.01) and 43.0% (95%CI: 28.6%–58.1%; *I*^*2*^ = 89.5%; *P*<0.01) (S5 Appendix), respectively. In females, the osteoporosis prevalence in 50–59, 50–69, and 70–79 was 27.8%, 60.4%, 75.4% (Fig 3). In males, the prevalence of osteoporosis in 50–59, 50–69, and 70–79 was 18.9%, 17.4%, 26.1% (Fig 3).

### Continent

**Fig 4:**
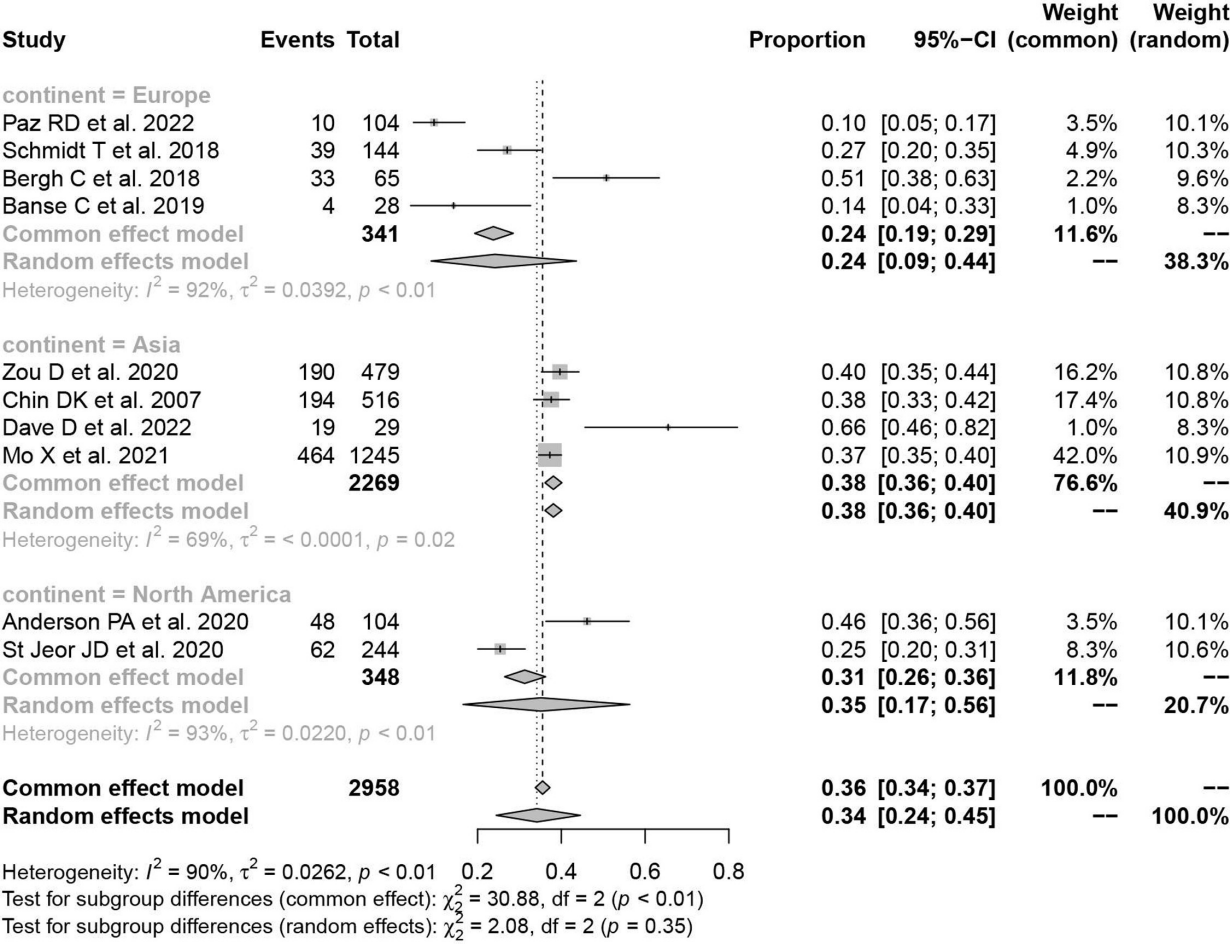
Forest plot of prevalence of osteoporosis in different continents.

Ten studies from three continents were included in our study. The prevalence of osteoporosis in Europe, Asia, and North America was 24.2% (95%CI: 8.9%–43.6%; *I*^*2*^ = 92.0%; *P*<0.01), 38.1% (95%CI: 36.1%–40.1%; *I*^*2*^ = 69.1%; *P*<0.01), 35.1% (95%CI: 16.5%–56.3%; *I*^*2*^ = 92.8%; *P*<0.01), respectively (Fig 4).

### Diagnoses classification

**Fig 5:**
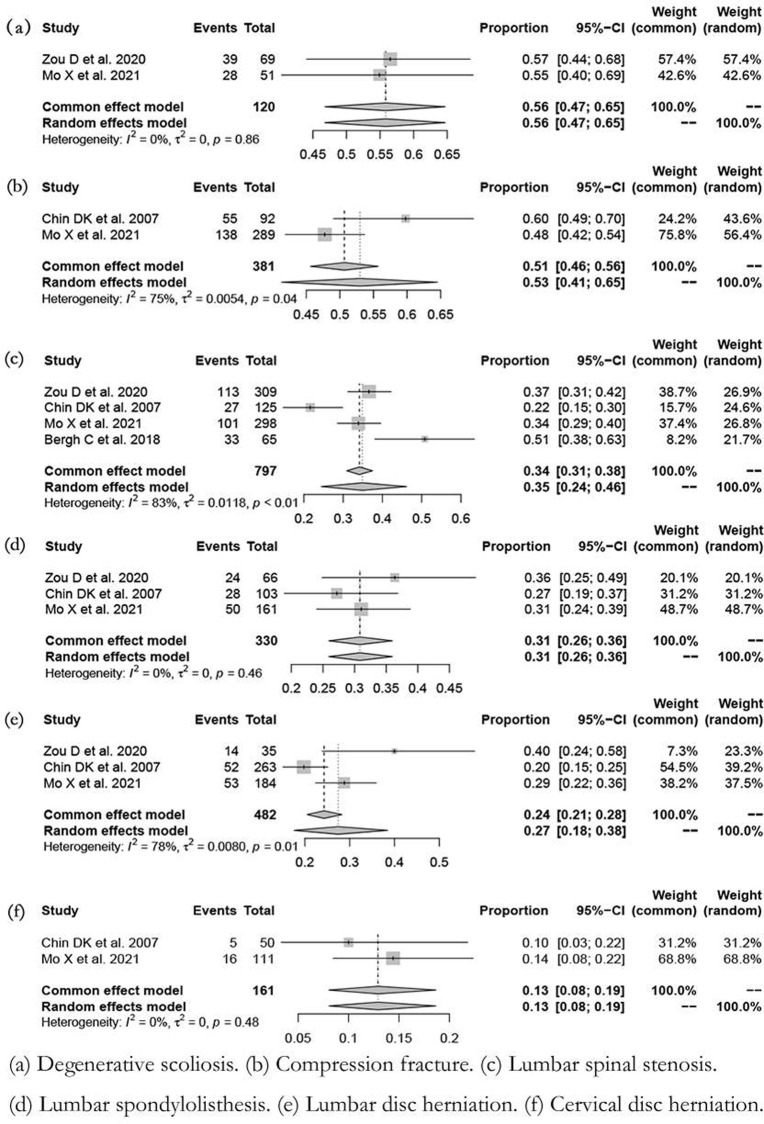
Forest plot of prevalence of osteoporosis in spine surgery based on diagnoses classification. (a) Degenerative scoliosis. (b) Compression fracture. (c) Lumbar spinal stenosis. (d) Lumbar spondylolisthesis. (e) Lumbar disc herniation. (f) Cervical disc herniation.

The osteoporosis prevalence is different based on different diagnosis classifications (Fig 5). The highest prevalence occurred in patients with lumbar scoliosis (55.8%; 95%CI: 46.8%-64.7%; *I*^2^ = 0%; *P* = 0.86) (Fig 5A), and the lowest occurred in patients with cervical disc herniation (12.9%; 95%CI: 8.1%-18.7%; *I*^2^ = 0%; *P* = 0.48) (Fig 5F). In patients with a compression fracture, lumbar spinal stenosis, lumbar spondylolisthesis, and lumbar disc herniation were 53.0%, 34.9%, 30.8%, and 27.4%, respectively (Fig 5B–5E).

#### Meta-regression analyses

The results of meta-regression analysis indicated that sample size (*P* = 0.81), quality score (*P* = 0.99), number of female participants (*P* = 0.82), publication year (*P* = 0.92), mean age of participants (*P* = 0.93), and study design (*P* = 0.77) were not contributed to the overall heterogeneity (S6 Appendix).

#### Sensitivity analysis and publication bias

After sensitivity analysis by eliminating individual studies, the overall osteoporosis prevalence varied from 31.6% (95%CI: 22.9–41.0%) to 37.4% (95%CI: 29.2–45.8%), and the *I*^*2*^ statistic values varied from 83.5% to 91.2%. The result of the funnel plot indicated the asymmetry between studies (S7 Appendix). However, Egger’s test (*P* = 0.61) indicated no publication bias.

## Discussion

Our systematic review and meta-analysis showed a high prevalence of osteoporosis in spinal surgery patients. In total, the prevalence of osteoporosis, osteopenia, and osteoporosis/osteopenia in patients undergoing spine surgery is 34.2%, 43.5%, and 78.7%, respectively. The prevalence of osteoporosis is significantly higher in females (43.0%) than in males (19.9%). A recommendation by the International Society for Clinical Densitometry (ISCD) suggested that surgeons should evaluate bone health in males aged ≥70 years and females aged ≥65 years who undergo spine surgery [15]. However, our study showed an unexpectedly high osteoporosis prevalence in patients aged under these cut-offs. In age groups 50–59, 60–69, and 70–79, the prevalence of osteoporosis is 27.8%, 60.4%, and 75.4% in females and 18.9%, 17.4%, and 26.1% in males. Considering the high osteoporosis rate and its associated surgery complication, the current osteoporosis screening and treatment before spine surgery may not be inadequate and need to raise awareness among orthopedic specialists in the future.

In our study, the prevalence of osteoporosis was higher than in some previous studies that investigated ordinary people [2, 27]. A large population-based meta-analysis showed that the global prevalence of osteoporosis was 14.1%, 31.8%, 50.9% for females, and 10.3%, 12.9%, 22.6% for males in 50–59, 60–69, and 70–79 years [2]. The prevalence of osteoporosis in our study varied by different procedures. The prevalence is highest in patients with degenerative scoliosis (55.8%). The lowest prevalence of osteoporosis occurs in patients with cervical disc herniation (12.9%). This finding is consistent with the previous research by Pappou et al. [28], which indicated that degenerative scoliosis is an important predictor of osteoporosis. One explanation may be that patients with osteoporosis are more prone to deformation of the weaker vertebrae in case of asymmetric loading caused by degenerative facet joints and the lumbar disc [29]. Another possible reason is that lumbar degenerative diseases such as scoliosis can cause considerable restriction of activities, which is associated with bone loss and osteoporosis [30]. In contrast, most types of cervical disc herniation usually do not experience the activity restriction. Another non-negligible reason for a higher osteoporosis rate in degenerative scoliosis patients is that they are usually older than other patients, such as those with cervical disc herniation. Previous studies have shown that degenerative scoliosis usually starts around the age of 50, and the average age of these patients is 70.5 years [31]. Other spinal disorders have also been reported to be associated with a risk of osteoporosis. A study by Kim et al. [32] showed a higher bone turnover rate in patients with spinal stenosis. In a case-control study, Park et al. [33] suggested that patients with spinal stenosis are less likely to benefit from ibandronate treatment compared with the control group. The neurological claudication caused by spinal stenosis could lead to reduced strength in the lower extremities and a higher bone loss rate [34]. In addition, a large retrospective study showed that patients with untreated spinal cord cervical spondylosis had 1.59 times the risk of fracture compared to general population controls [35]. Lumbar spondylolisthesis may be associated with spinal curvatures, such as thoracic kyphosis, which is an important alternative osteoporosis marker [36, 37]. These direct or indirect factors may lead to a high osteoporosis rate in patients with spinal disorders. However, the pathological mechanisms contributing to the tendency of osteoporosis, such as its biomechanical alterations [38], need to be further investigated.

Our results showed the prevalence of osteoporosis in females (43.0%) is significantly higher than in males (19.9%), in line with the previous findings [39]. We found that female patients are more likely to be affected by age. In the 50–59 age group, the osteoporosis prevalence was slightly higher in women than in men (27.8% vs. 18.9%). However, In the 70–79 age group, the prevalence was much higher in women than in men (75.4% vs. 26.1%), and more than three-quarters of female patients had osteoporosis. Postmenopausal estrogen deficiency and differences in the distribution of factors like diabetes, obesity, and metabolic syndrome between the gender may account for this difference [40–42]. The subgroup analysis in the study showed that the previous was highest in Asia (38.1%) and lowest in Europe (24.2%). Some previous studies observed that Asians have lower BMD than Europeans, which could be explained by the smaller body and bone size of Asians [43, 44]. Nevertheless, more studies are needed to validate our results due to the potential heterogeneity led by the small number of enrolled studies.

Bone health in orthopedic surgery has attracted wide attention since it may influence the outcome of the surgery [45–47]. In joint arthroplasty [45], osteoporosis will impair osseointegration and lead to failed surgery. Similarly, osteoporosis in spine surgery has raised concerns recently, as successful spine surgery requires adequate BMD for proper fixation strength, long-term stability, and lower instrumentation failure risk [48]. In a retrospective study in America, DeWald et al. [49] investigated the early and late complications in osteoporosis patients who received lumbar fusions. They found that 13% of participants experienced early complications (under 3 months), including epidural hemaoma and adjacent compression fractures. Furthermore, late postoperative complications including pseudoarthroses with rod breakage (11%), disc herniation (4%), instrumentation loosening (7%), instrumentation (11%), and proximal junctional kyphosis (26%) [49]. In cervical spine surgery, Guzman et al. revealed that patients with osteoporosis experienced a higher risk of postoperative hemorrhage (OR: 1.70), a higher risk of revision (OR: 1.54) and a longer hospitalization time and costs [50]. Therefore, perioperative management has been widely used in osteoporotic patients, including pharmacological therapy, cement augmentation of pedicle screws, multiple points fixations, and the newly designed pedicle screw [10, 51]. In a prospective randomized trial, Nagahama et al. [52] found that patients who received an alendronate treatment had higher fusion rates and lowered cage subsidence rates compared with control group after posterior lumbar interbody fusion. Surgeons have widely discussed surgical techniques in the osteoporosis spine. Guo et al. [10] reported that selective cement augmentation of cranial and caudal pedicle screws could provide comparable stability for osteoporosis patients. Cement screws have been considered appropriate for osteoporotic spine [53]. The management of osteoporosis for patients undergoing elective spine surgeries should be a concern for orthopedic surgeons. Experts recommend that all patients undergoing elective spine surgery have adequate preoperative vitamin D and calcium status [54]. In addition, bone anabolic pharmaceuticals like abaloparatide or teriparatide are recommended as the first-line treatment for osteoporosis patients undergoing spine surgery if there are no contraindications [54]. However, several previous studies have reported insufficient preoperative bone health screening for spinal surgery [13, 55]. Díaz-Romero et al. showed that 32.5% of surgeons would not consider a bone health assessment before the spinal arthrodesis, and 37.7% of surgeons would not consider an osteoporosis treatment before and after treatment [55]. A survey of 114 surgeons revealed that only 60% of surgeons would consider a preoperative bone health assessment for patients who experienced a low-energy spine fracture. The proportion dropped to 44% for patients with instrumented fusion [13]. Thus, the high osteoporosis rate in spine surgery should be a major concern for spine surgeons, and bone health in patients undergoing spinal surgery should be appropriately screened and optimized.

Our study has some limitations. First, we included only DXA as a criterion for the osteoporosis diagnosis due to the original study data limitations. In recent years, the vertebral body HU values estimated from CT scans have been extensively studied for the assessment of osteoporosis [13, 56, 57]. Zou et al. reported a 74.1% diagnosis specificity of DXA compared with CT scans in patients with degenerative diseases. They explain that the CT HU is less affected by lumbar degeneration by avoiding the degenerative regions [18]. However, there has been no consensus on the specific HU values for diagnosing osteoporosis [56, 58]. More serious radiation damage from CT compared to DXA is also an essential factor in physician decision-making. Whether the CT scan for osteoporosis is better than DXA has also not been proven and needs further investigation [59, 60]. Second, osteoporosis was defined differently based on the femur or lumbar spine in the original studies. Previous studies have proven that the osteoporosis rate was different based on these sites in the same cohort [61]. Third, only ten studies and 2,958 participants were included in our analysis, which could increase the heterogeneity. Fourth, we included studies from different countries, which may increase heterogeneity because of the different backgrounds of the populations. Fifth, the overall heterogeneity across the studies was high. More studies with a larger sample and stronger evidence should be conducted to explore the association between osteoporosis and lumbar surgery.

## Conclusion

Our results showed a high prevalence of osteoporosis in patients undergoing spine surgery, especially in females, people of older age, and patients who received degenerative scoliosis and compression fractures. Current osteoporosis screening standards for patients undergoing spine surgery may not be adequate. Orthopedic specialists should make more efforts regarding preoperative osteoporosis screening and treatment.

## Supporting information

S1 Appendix PRISMA 2009 checklist. (DOCX)

S2 Appendix Detailed search strategy in three databases. (DOCX)

S3 Appendix Risk of bias tool for prevalence studies. (DOCX)

S4 Appendix The checklist of prevalence study quality. (DOCX)

S5 Appendix Forest plot of prevalence of female (a) and male (b) in patients undergoing spine surgery.
(TIF)

S6 Appendix Meta-regression analyses of the effects of potential moderators on overall heterogeneity. (DOCX)

S7 Appendix The funnel plots for included studies of prevalence of osteoporosis in patients undergoing spine surgery. (TIF)

## List of abbreviations

BMD: Bone mineral density
DXA: Dual-energy Xray absorptiometry
ISCD: International Society for Clinical Densitometry
PRISMA: Preferred Reporting Items for Systematic and Meta-analyses guidelines
