# Supplementary material for: Prevalence of osteoporosis in spinal surgery patients older than 50 years: A systematic review and meta-analysis
Source: PLoS One. 2023 May 25;18(5):e0286110. doi: 10.1371/journal.pone.0286110 (PMC10212156; doi:10.1371/journal.pone.0286110)
Supplement: S2 Appendix — (DOCX) [file pone.0286110.s002.docx]

**Appendix 1.1 Search strategy in PUBMED**

| **No.** | **Query** | **Results** |
| --- | --- | --- |
| 4 | #1 AND #2 AND #3 | [625](https://pubmed.ncbi.nlm.nih.gov/?term=%28%28%28%28%28lumbar+surgery%5BMeSH+Terms%5D%29+OR+%28spine+surgery%5BMeSH+Terms%5D%29%29+OR+%28lumbar+surgery%5BMeSH+Terms%5D%29%29+OR+%28spine+surgery%5BMeSH+Terms%5D%29%29+AND+%28%28%28%28%28%28osteoporosis%5BMeSH+Terms%5D%29+OR+%28osteopenia%5BMeSH+Terms%5D%29%29+OR+%28bone+mineral+density%5BMeSH+Terms%5D%29%29+OR+%28osteoporosis%5BTitle%2FAbstract%5D%29%29+OR+%28osteopenia%5BTitle%2FAbstract%5D%29%29+OR+%28bone+mineral+density%5BTitle%2FAbstract%5D%29%29%29+AND+%28%28%28%28%28%28prevalence%5BMeSH+Terms%5D%29+OR+%28incidence%5BMeSH+Terms%5D%29%29+OR+%28epidemiology%5BMeSH+Terms%5D%29%29+OR+%28prevalence%5BTitle%2FAbstract%5D%29%29+OR+%28incidence%5BTitle%2FAbstract%5D%29%29+OR+%28epidemiology%5BTitle%2FAbstract%5D%29%29&sort=relevance) |
| 3 | **(((((prevalence[MeSH Terms]) OR (incidence[MeSH Terms])) OR (epidemiology[MeSH Terms])) OR (prevalence[Title/Abstract])) OR (incidence[Title/Abstract])) OR (epidemiology[Title/Abstract])** | [1,883,340](https://pubmed.ncbi.nlm.nih.gov/?term=%28%28%28%28%28prevalence%5BMeSH+Terms%5D%29+OR+%28incidence%5BMeSH+Terms%5D%29%29+OR+%28epidemiology%5BMeSH+Terms%5D%29%29+OR+%28prevalence%5BTitle%2FAbstract%5D%29%29+OR+%28incidence%5BTitle%2FAbstract%5D%29%29+OR+%28epidemiology%5BTitle%2FAbstract%5D%29&sort=relevance) |
| 2 | **(((((osteoporosis[MeSH Terms]) OR (osteopenia[MeSH Terms])) OR (bone mineral density[MeSH Terms])) OR (osteoporosis[Title/Abstract])) OR (osteopenia[Title/Abstract])) OR (bone mineral density[Title/Abstract])** | [155,807](https://pubmed.ncbi.nlm.nih.gov/?term=%28%28%28%28%28osteoporosis%5BMeSH+Terms%5D%29+OR+%28osteopenia%5BMeSH+Terms%5D%29%29+OR+%28bone+mineral+density%5BMeSH+Terms%5D%29%29+OR+%28osteoporosis%5BTitle%2FAbstract%5D%29%29+OR+%28osteopenia%5BTitle%2FAbstract%5D%29%29+OR+%28bone+mineral+density%5BTitle%2FAbstract%5D%29&sort=relevance) |
| 1 | **(((lumbar surgery[MeSH Terms]) OR (spine surgery[MeSH Terms])) OR (lumbar surgery[MeSH Terms])) OR (spine surgery[MeSH Terms])** | [90,757](https://pubmed.ncbi.nlm.nih.gov/?term=%28%28%28lumbar+surgery%5BMeSH+Terms%5D%29+OR+%28spine+surgery%5BMeSH+Terms%5D%29%29+OR+%28lumbar+surgery%5BMeSH+Terms%5D%29%29+OR+%28spine+surgery%5BMeSH+Terms%5D%29&sort=relevance) |

**Appendix 1.2 Search strategy in EMBASE**

| **No.** | **Query** | **Results** |
| --- | --- | --- |
| #4 | #1 AND #2 AND #3 | 1,133 |
| #3 | 'prevalence'/exp OR 'incidence'/exp OR 'epidemiology'/exp OR prevalence:ab,ti OR incidence:ab,ti OR epidemiology:ab,ti | 5,148,200 |
| #2 | 'osteoporosis'/exp OR 'osteopenia'/exp OR 'bone mineral dnsity' OR osteoporosis:ab,ti OR osteopenia:ab,ti OR 'bone mineral dnsity':ab,ti | [180,723](https://www.embase.com/) |
| #1 | 'lumbar surgery'/exp OR 'spine surgery'/exp OR 'lumbar surgery':ab,ti OR 'spine surgery':ab,ti | 106,524 |

**Appendix 1.3 Search strategy in SCOPUS**

| **No.** | **Query** | **Results** |
| --- | --- | --- |
| 4 | #1 AND #2 AND #3 | [1,178](https://www.webofscience.com/wos/alldb/summary/a6ec982f-5611-4dd1-9fe1-acef8f4e1fa2-50bc6dd4/relevance/1) |
| 3 | **((TS=(osteoporosis)) OR TS=(osteopenia)) OR TS=(bone mineral density)** | [259,263](https://www.webofscience.com/wos/alldb/summary/d9bcd64d-9808-434d-9b48-de71c474616c-50bc3943/relevance/1) |
| 2 | **((TS=(osteoporosis)) OR TS=(osteopenia)) OR TS=(bone mineral density)** | [259,263](https://www.webofscience.com/wos/alldb/summary/d9bcd64d-9808-434d-9b48-de71c474616c-50bc3943/relevance/1) |
| 1 | (TS=(lumbar surgery')) OR TS=(spine surgery) | [125,810](https://www.webofscience.com/wos/alldb/summary/53c293bc-75f6-4b08-a5d1-6ac8fd365b9e-50bc2541/relevance/1) |
