# Supplementary material for: Prevalence of osteoporosis in spinal surgery patients older than 50 years: A systematic review and meta-analysis
Source: PLoS One. 2023 May 25;18(5):e0286110. doi: 10.1371/journal.pone.0286110 (PMC10212156; doi:10.1371/journal.pone.0286110)
Supplement: S3 Appendix — (DOCX) [file pone.0286110.s003.docx]

Appendix 3. Risk of bias tool for prevalence studies

| Item |
| --- |
| External validity |
| 1. Was the study’s target population a close representation of the national population in relation to relevant variables? |
| 2. Was the sampling frame a true or close representation of the target population? |
| 3. Was some form of random selection used to select the sample, OR was a census undertaken? |
| 4. Was the likelihood of nonresponse bias minimal? |
| Internal validity |
| 5. Were data collected directly from the subjects (as opposed to a proxy)? |
| 6. Was an acceptable case definition used in the study? |
| 7. Was the study instrument that measured the parameter of interest shown to have validity and reliability? |
| 8. Was the same mode of data collection used for all subjects? |
| 9. Was the length of the shortest prevalence period for the parameter of interest appropriate? |
| 10. Were the numerator(s) and denominator(s) for the parameter of interest appropriate? |
| 11. Summary item on the overall risk of study bias |
