# Supplementary material for: Prevalence of osteoporosis in spinal surgery patients older than 50 years: A systematic review and meta-analysis
Source: PLoS One. 2023 May 25;18(5):e0286110. doi: 10.1371/journal.pone.0286110 (PMC10212156; doi:10.1371/journal.pone.0286110)
Supplement: S4 Appendix — (DOCX) [file pone.0286110.s004.docx]

**Appendix 4:** The Checklist of Prevalence Study Quality.

| Author(et.al), Year | 1. Was the study’s target population a close representation of the national population in relation to relevant variables? | 2. Was the sampling frame a true or close representation of the target population? | 3. Was some form of random selection used to select the sample, OR was a census undertaken? | 4. Was the likelihood of nonresponse bias minimal? | 5. Were data collected directly from the subjects (as opposed to a proxy)? | 6. Was an acceptable case definition used in the study? | 7. Was the study instrument that measured the parameter of interest shown to have validity and reliability? | 8. Was the same mode of data collection used for all subjects?d | 9. Was the length of the shortest prevalence period for the parameter of interest appropriate? | 10. Were the numerator(s) and denominator(s) for the parameter of interest appropriate? | 11. Summary item on the overall risk of study bias |
| --- | --- | --- | --- | --- | --- | --- | --- | --- | --- | --- | --- |
| Paz RD et al. 2022 | 1 | 1 | 1 | 0 | 0 | 1 | 1 | 1 | 1 | 0 | 8 |
| Schmidt T et al. 2018 | 1 | 1 | 1 | 1 | 0 | 1 | 1 | 1 | 1 | 0 | 9 |
| Bergh C et al. 2018 | 1 | 1 | 1 | 0 | 0 | 1 | 1 | 1 | 1 | 0 | 8 |
| Banse C et al. 2019 | 1 | 1 | 1 | 0 | 0 | 0 | 1 | 0 | 1 | 0 | 6 |
| Zou D et al. 2020 | 1 | 1 | 1 | 1 | 0 | 1 | 1 | 0 | 1 | 0 | 8 |
| Chin DK et al. 2007 | 1 | 1 | 1 | 1 | 0 | 1 | 1 | 1 | 1 | 0 | 9 |
| Dave D et al. 2022 | 1 | 1 | 0 | 0 | 1 | 1 | 1 | 0 | 1 | 0 | 7 |
| Mo X et al. 2021 | 1 | 1 | 1 | 1 | 0 | 1 | 1 | 1 | 1 | 0 | 9 |
| Anderson PA et al. 2020 | 1 | 1 | 1 | 1 | 0 | 1 | 1 | 0 | 1 | 0 | 8 |
| St Jeor JD et al. | 1 | 1 | 1 | 1 | 0 | 1 | 1 | 1 | 1 | 0 | 9 |
