# Supplementary figures and images for: Prevalence of osteoporosis in spinal surgery patients older than 50 years: A systematic review and meta-analysis
Source: PLoS One. 2023 May 25;18(5):e0286110. doi: 10.1371/journal.pone.0286110 (PMC10212156; doi:10.1371/journal.pone.0286110)

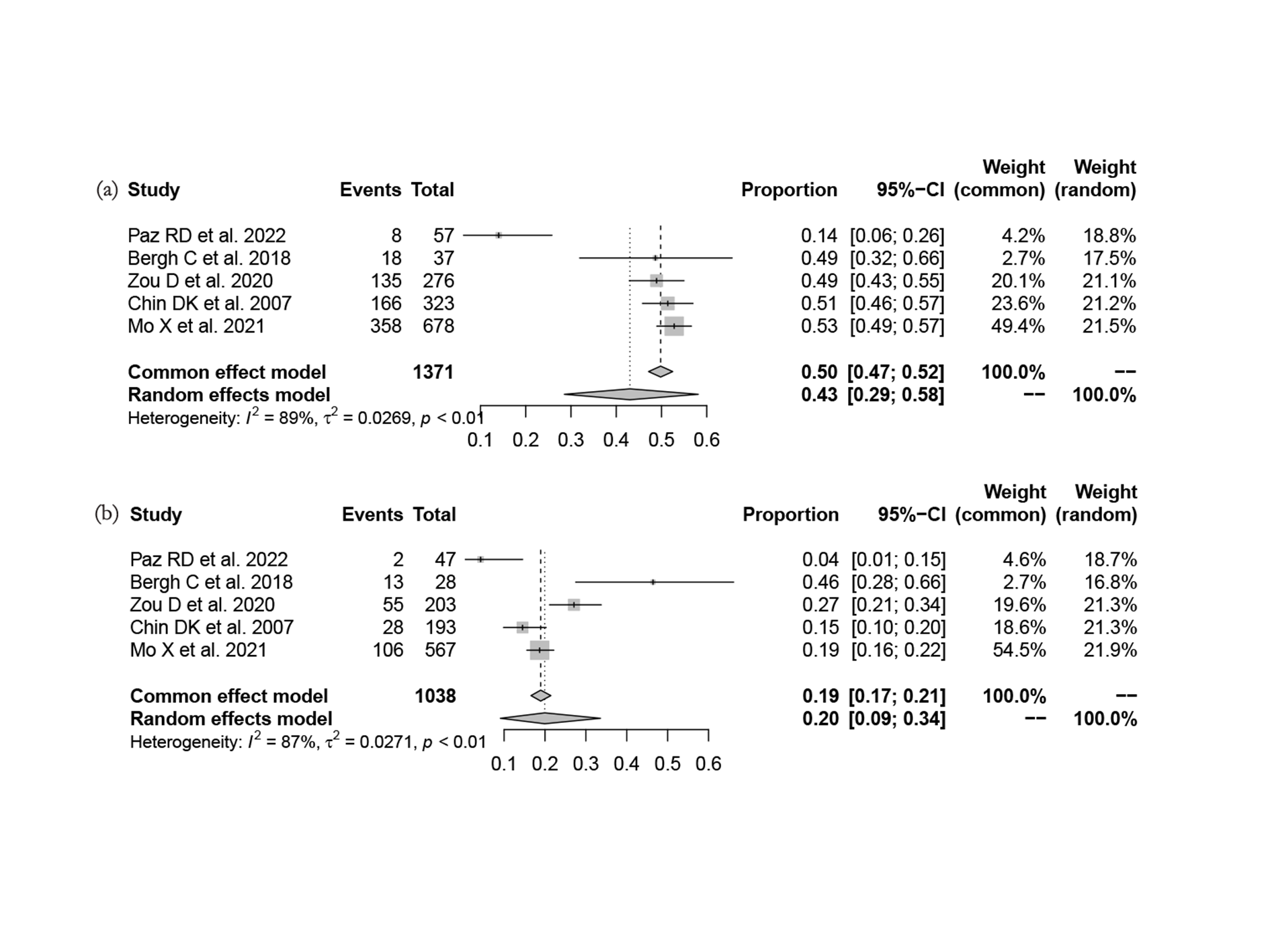

Supplement: S5 Appendix — Forest plot of prevalence of female (a) and male (b) in patients undergoing spine surgery. (TIF) [file pone.0286110.s005.tif]

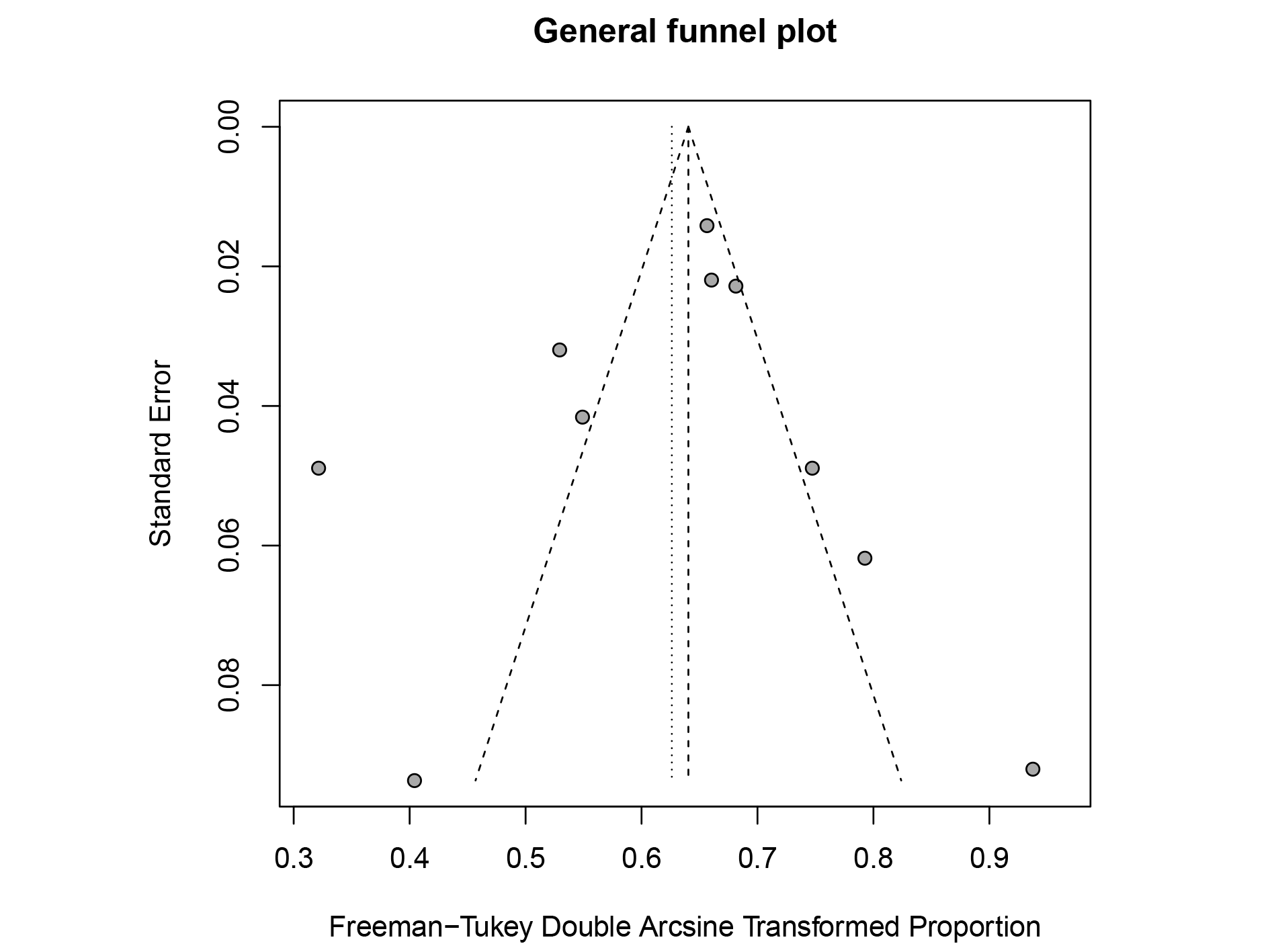

Supplement: S7 Appendix — (TIF) [file pone.0286110.s007.tif]
