# Supplementary material for: Prevalence of osteoporosis in spinal surgery patients older than 50 years: A systematic review and meta-analysis
Source: PLoS One. 2023 May 25;18(5):e0286110. doi: 10.1371/journal.pone.0286110 (PMC10212156; doi:10.1371/journal.pone.0286110)
Supplement: S6 Appendix — (DOCX) [file pone.0286110.s006.docx]

**Appendix 6:** Meta-regression analyses of the effects of potential moderators on overall heterogeneity.

|  | **Z value** | **95%CI.lb** | **95%CI.ub** | ***P* value** |
| --- | --- | --- | --- | --- |
| **Sample size** | **0.2403** | **-0.0088** | **0.0112** | **0.8101** |
| **Female numbers** | **-0.2223** | **-0.0207** | **0.0165** | **0.8241** |
| **Quality score** | **-0.0093** | **-0.2862** | **0.2835** | **0.9926** |
| **Publication year** | **-0.1064** | **-0.0686** | **0.0615** | **0.9153** |
| **Mean age** | **0.0919** | **-0.0657** | **0.0722** | **0.9268** |
| **Study design** | **0.2861** | **-0.3446** | **0.4624** | **0.7748** |
